# Supplementary material for: Converting histidine-induced 3D protein arrays in crystals into their 3D analogues in solution by metal coordination cross-linking
Source: Commun Chem. 2020 Nov 2;3:151. doi: 10.1038/s42004-020-00394-x (PMC9814774; doi:10.1038/s42004-020-00394-x)
Supplement: Supplementary file 1 — Supplementary Information [file 42004_2020_394_MOESM1_ESM.pdf]

Supporting Information

for

Converting histidine-induced 3D protein arrays in crystals into their 3D analogues in solution by metal coordination cross-linking

Xiaoyi Tan[1]*, Hai Chen*[1]*, Chunkai Gu*[1]*, Jiachen Zang*[1]*, Tuo Zhang*[1]*, Hongfei Wang*[2]*, Guanghua Zhao*[1,*]

[1]College of Food Science & Nutritional Engineering, China Agricultural University, Key Laboratory of Functional Dairy, Ministry of Education, Beijing 100083, China.

[2]Key Laboratory of Chemical Biology and Molecular Engineering of Education Ministry, Institute of Molecular Science, Shanxi University, Taiyuan 030006, China

*Corresponding author: Guanghua Zhao, E-mail: gzhao@cau.edu.cn, Phone: 0086-10-62738737

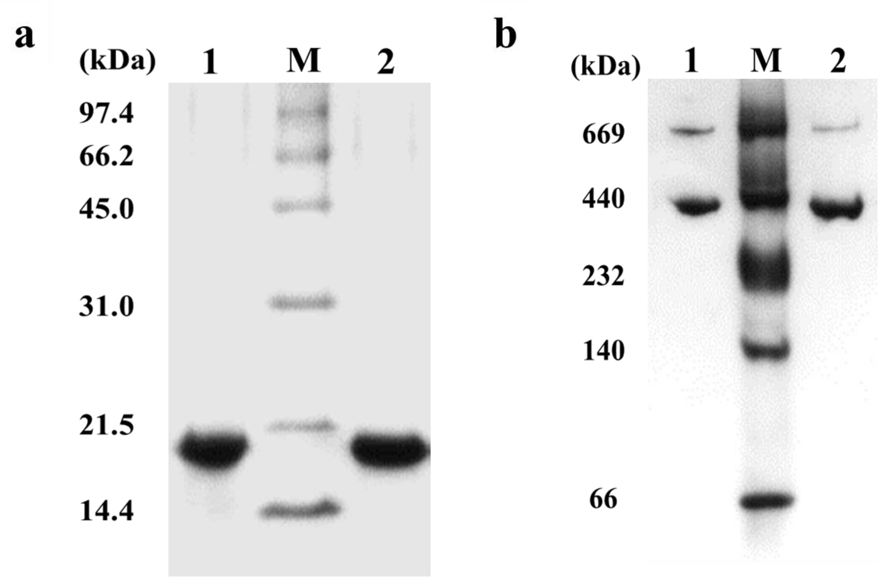

Supplementary Figure 1. Preparation and characterization of ^{T158H}MjFer. a SDS-PAGE and **b** Native PAGE analyses of purified MjFer and ^{T158H}MjFer. Lane M, protein makers; lane 1, MjFer; lane 2, ^{T158H}MjFer.

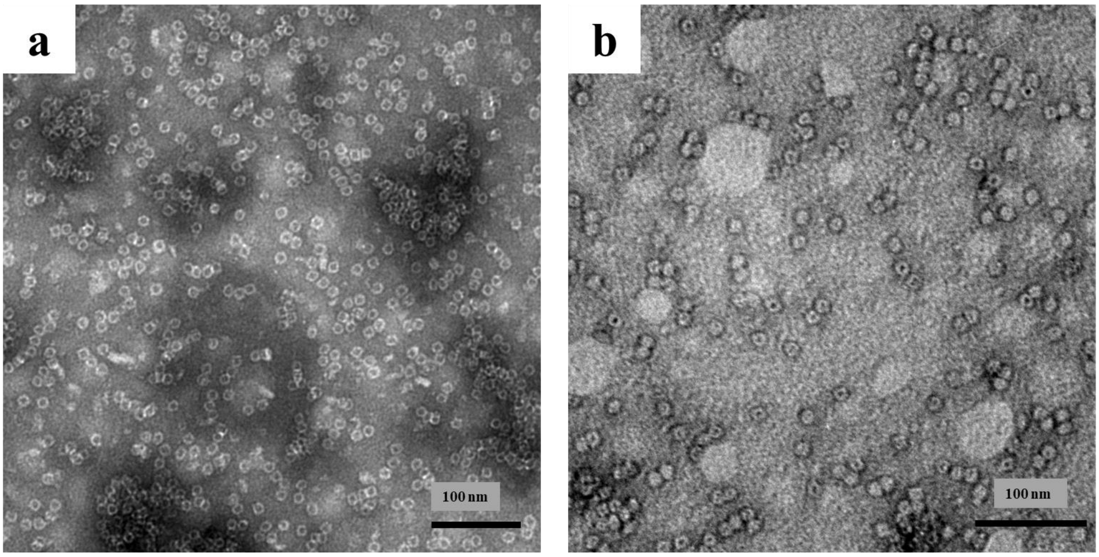

Supplementary Figure 2. TEMs images of ferritin in salt-free buffer. a TEM image of 1.0 µM wild-type MjFer in 25 mM Tris-HCl, pH 8.0. **b** TEM image of 1.0 µM ^{T158H}MjFer in the buffer of 25 mM Tris-HCl, pH 8.0.

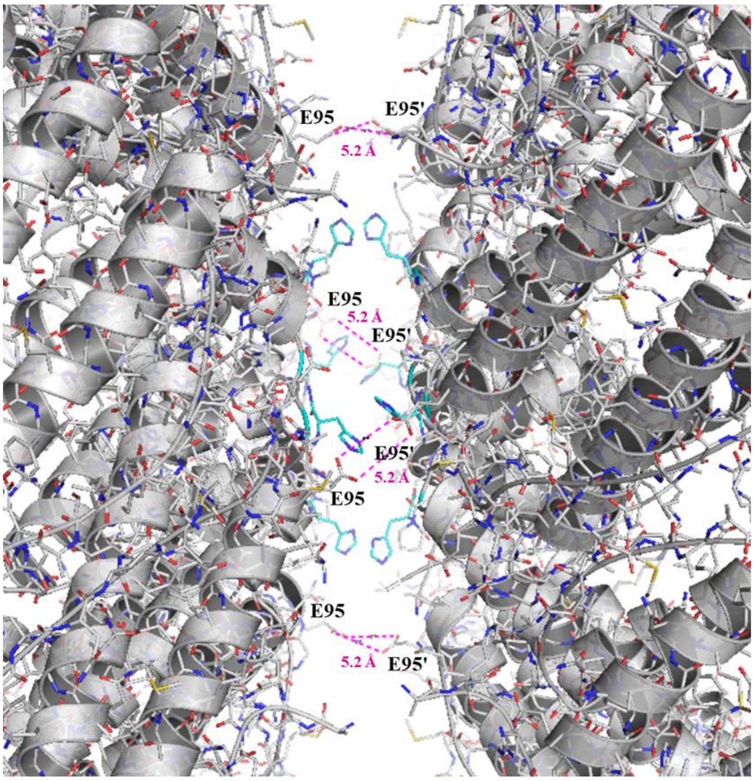

Supplementary Figure 3. A side view of the protein-protein interface of ^{T158H}MjFer. The dash lines show that the distance between four pairs of glutamic acid residues is the closest.

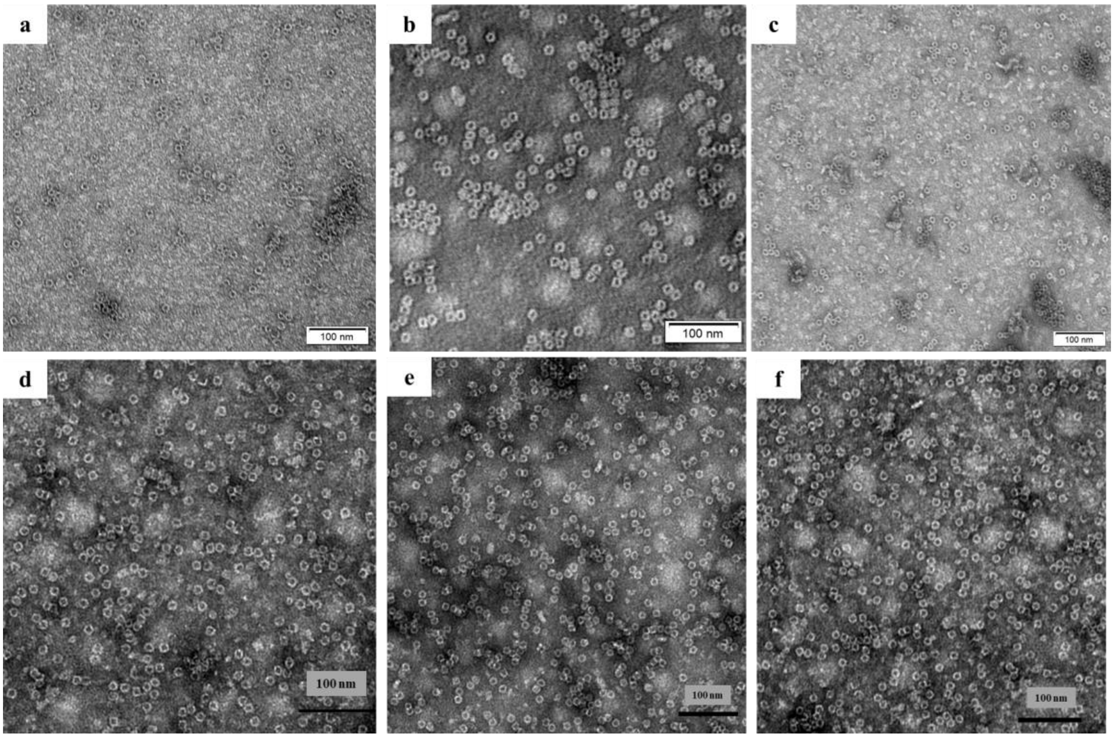

Supplementary Figure 4. **TEM images of $^{T158H/E95A}$MjFer at different salt and nickel ions concentrations. a-c** TEM image of 1.0 µM $^{T158H/E95A}$MjFer at 25 mM Tris-HCl pH 8.0 buffer containing 0.7 mM $NiSO_4$, 500 mM NaCl and 0.7 mM Ni^{2+} plus 500 mM NaCl, respectively. **d-f** TEM image of 1.0 µM wild-type MjFer at 25 mM Tris-HCl pH 8.0 buffer containing 500 mM NaCl plus 0.7 mM $NiSO_4$.

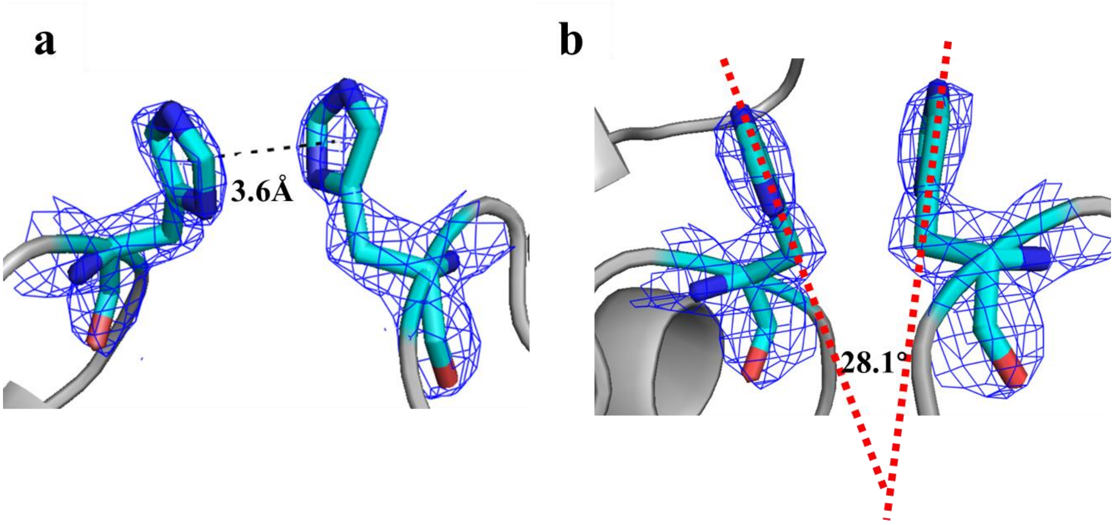

Supplementary Figure 5. Close-up views of the His-His interactions in the crystal structure of Ni^{2+}-induced 3D ^{T158H}MjFer arrays. a A side view shows that the distance between two imidazole group rings at protein-protein interfaces is about 3.6 Å. **b** The angle between two imidazole rings from a pair of His residues is about 28.1°. Blue mesh is the electron density at $\sigma = 3$.

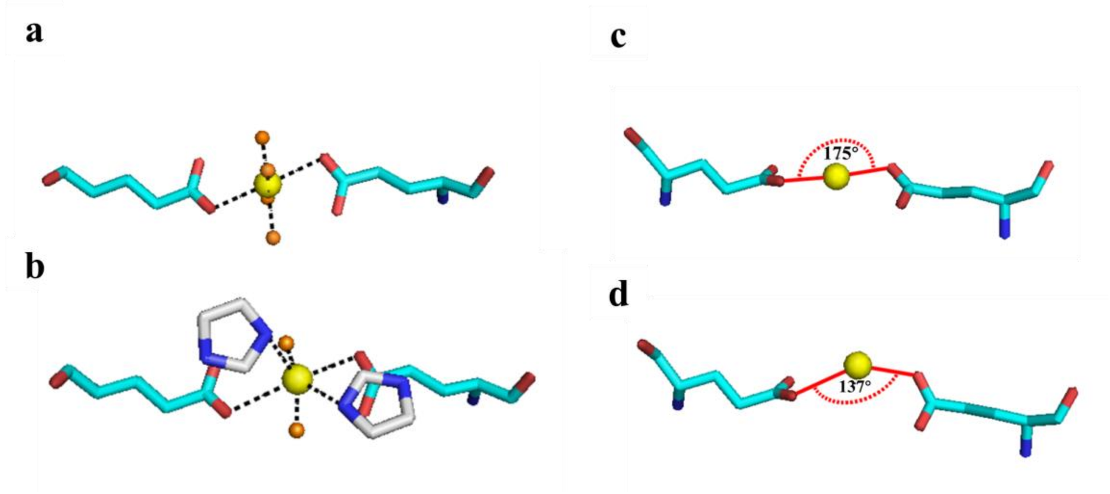

Supplementary Figure 6. Comparison of the crystal structure of ^{T158H}MjFer-Ni^{2+}- H_2O complexes and ^{T158H}MjFer-Ni^{2+}- imidazole complex. **a** A closeup view of the coordination geometry of ^{T158H}MjFer-Ni^{2+}- H_2O complex. **b** A closeup view of the coordination geometry of ^{T158H}MjFer-Ni^{2+}- imidazole complex. **c** The angle of O_{Glu}-Ni^{2+}-O_{Glu} in the absence of imidazole is around 175°. **d** The angle of O_{Glu}-Ni^{2+}-O_{Glu} decreased to 137° after two coordinated H_2O molecules were replaced with two imidazole molecules.

Supplementary Table 1 Crystallization conditions for each crystal.

Proteins	Reservoir
^{T158H}MjFer (pH 4.0)	2000 mM NaCl, 100 mM KH_2PO_4/Na_2HPO_4 (pH 4.0)
^{T158H}MjFer (pH 7.0)	2500 mM NaCl, 100 mM KH_2PO_4/Na_2HPO_4 (pH 7.0)
^{T158H}MjFer (pH 9.0)	2500 mM NaCl, 100 mM KH_2PO_4/Na_2HPO_4 (pH 9.0)
^{T158H}MjFer + Ni^{2+}	2500 mM NaCl, 100 mM KH_2PO_4/Na_2HPO_4 (pH 8.0), 2 mM $NiSO_4$
^{T158H}MjFer + Ni^{2+}+ imidazole	2000 mM NaCl, 100 mM imidazole (pH 8.0), 2 mM $NiSO_4$

Supplementary Table 2 Crystallographic statistics.

**Parameters**	**^{T158H}MjFer (pH 4.0)**	**^{T158H}MjFer (pH 7.0)**	**^{T158H}MjFer (pH 9.0)**	**^{T158H}MjFer + Ni^{2+}**	**^{T158H}MjFer + Ni^{2+}+ imidazole**
Beamline	SSRF BL18U1	SSRF BL18U1	SSRF BL18U1	SSRF BL18U1	SSRF BL17U1
Wavelength (Å)	0.9785	0.97853	0.97853	0.979304	1.4813
Space group	*P23*	*P23*	*P23*	*P432*	*P432*
Unit cell	117.435, 117.435, 117.435,90, 90, 90	117.114, 117.114, 117.114, 90, 90, 90	117.922,117.922, 117.922, 90, 90, 90	117.186, 117.186, 117.186, 90, 90, 90	117.349, 117.349, 117.349, 90, 90, 90
[a]Resolution (Å)	23.49-1.60	47.81-2.402	48.14-2.302	26.20 -1.702	35.38-2.398
[a]Redundancy	19.1	19.1	19.7	36.1	34.3
[a]Completeness (%)	100.00	100.00	100.00	100.00	100.00
[a]$I/\sigma I$	6.85	19.56	43.08	25.14	32.75
[a,b]R_{merge}	0.130	0.146	0.109	0.199	0.237
[a,c]R_{pim}	0.030	0.033	0.025	0.033	0.042
[d]$CC_{1/2}$	0.844	0.965	0.982	0.851	0.995
Unique reflections	71150 (7060)	21203 (2098)	24517 (2407)	30753 (3012)	11391 (1109)
Number of Atoms	2991	2818	2819	1711	1576
Refinement/Protein	2724	2724	2724	1428	1373
Refinement/Ligands	0	0	0	3	13
Refinement/H_2O	267	94	95	280	190
[e]R_{work}/R_{free}	0.1750 (0.2175) / 0.1877 (0.2231)	0.1908 (0.2229) / 0.2365 (0.2623)	0.1907 (0.1842) / 0.2060 (0.2450)	0.1479 (0.2090) / 0.1735 (0.2586)	0.1305 (0.1072) / 0.1833 (0.1804)
Wilson B-factor (Å^2)	19.16	20.07	25.59	18.57	13.20
B-factors (Å^2)/Protein	19.63	23.33	27.55	18.69	16.84

B-factors (Å^2)/Ligands	0	0	0	30.12	37.93
B-factors (Å^2)/H_2O	26.91	17.50	25.03	37.34	25.38
Bond lengths (Å)	0.016	0.017	0.019	0.006	0.011
Bond angles (°)	1.68	1.76	1.88	0.78	1.021
Ramachandran plot (%)					
Favored	99	99	98	99	99.4
Allowed	0.9	1.2	1.8	1.1	0.6
Outliers	0	0	0	0	0

[a]Highest resolution shell is shown in parentheses.

[b]$R_{merge} = \sum_{hkl} \sum_j |I_j(hkl)-<I(hkl)>| / \sum_{hkl} \sum_j I_j(hkl)$, where I is the intensity of reflection.

[c]$R_{pim}=\sum_{hkl} [1/(N-1)]^{1/2} \sum_j |I_j(hkl)-<I(hkl)>| / \sum_{hkl} \sum_j I_j(hkl)$, where N is the redundancy of the dataset.

[d]$CC_{1/2}$ is the correlation coefficient of the half datasets.

[e]$R_{work} = \sum_{hkl} | |F_{obs}| - |F_{calc}| | / \sum_{hkl} |F_{obs}|$, where F_{obs} and F_{calc} is the observed and the calculated structure factor, respectively. R_{free} is the cross-validation R factor for the test set of reflections (5% of the total) omitted in model refinement.
